# Effects of intravenous anesthesia with sevoflurane combined with propofol on intraoperative hemodynamics, postoperative stress disorder and cognitive function in elderly patients undergoing laparoscopic surgery

**DOI:** 10.12669/pjms.38.7.5763

**Published:** 2022

**Authors:** Li Yang, Zhigang Chen, Dengguo Xiang

**Affiliations:** 1 Li Yang, Department of Anesthesiology, Xianning Central Hospital, The First Affiliated Hospital of Hubei University of Science and Technology, Xianning 437000, Hubei, P.R. China; 2 Zhigang Chen, Department of Anesthesia and Pain, Wuhan Xinzhou District People’s Hospital, Wuhan 430400, Hubei, P.R. China; 3 Dengguo Xiang, Department of Anesthesiology, The Central Hospital of Enshi Tujia and Miao Autonomous Prefecture, Enshi 445000, Hubei, P.R. China

**Keywords:** Intravenous anesthesia, sevoflurane combined with propofol, Laparoscopic surgery, Intraoperative hemodynamics, Postoperative stress disorder, Cognitive function

## Abstract

**Objectives:**

To analyze the effects of intravenous anesthesia with sevoflurane combined with propofol on intraoperative hemodynamics, postoperative stress disorder and cognitive function in elderly patients undergoing laparoscopic surgery.

**Methods:**

Eighty elderly patients undergoing laparoscopic surgery in Xianning Central Hospital from September 2014 to February 2018. Enrolled patients were divided into observation group (n=40) and control group (n=40) according to the random regionalization method. Patients in the control group were given intravenous anesthesia with propofol, while those in the observation group were provided with a combined use of sevoflurane based on the method in the control group. The general surgical data, intraoperative hemodynamics, postoperative stress disorder and cognitive function were compared between the two groups.

**Results:**

There were no blood transfusions and no complications during operation in the two groups, and the operation completed successfully. There was no significant difference in the time of unconsciousness, intubation time, and duration of pneumoperitoneum establishment (p>0.05). However, the spontaneous breathing time, eye opening time upon calling and extubation time in observation group were shorter than those in control group (p<0.05). The heart rate (HR) in the two groups at intubation and at exploration was significantly higher than that at the time of entering the room (p<0.05), which was lower in observation group than that in control group (p<0.05). While there was no significant difference in the index at the other time points compared with that before entering the room and control group (p>0.05). The systolic blood pressure (SBP) had different degrees of change before intubation, after 0 minutes, three minutes and six minutes of intubation, after 0 minutes and five minutes of pneumoperitoneum establishment and at exploration when compared with that before entering the room (p<0.05). But the SBP at intubation and at exploration was significantly lower in observation group than that in control group (p<0.05). MAP was only decreased significantly at intubation compared with that before entering the room (p<0.05). At exploration, there was no significant fluctuation in the MAP level in observation group (p>0.05), but the MAP was increased significantly in control group (p<0.05). In addition, the postoperative stress disorder in observation group was milder than that in control group (p<0.05), and the postoperative cognitive function was better than that in control group (p<0.05).

**Conclusions:**

Intravenous anesthesia with sevoflurane combined with propofol has little effects on intraoperative hemodynamics and postoperative cognitive function in elderly patients undergoing laparoscopic surgery. Besides, it can obtain better recovery quality and have milder postoperative stress disorder than single use of propofol.

## INTRODUCTION

There is an extensive application of laparoscopic surgery in clinical practice with the increasing development of minimally invasive technology.^1^,^2^ Compared with traditional open surgery, laparoscopic surgery has exhibited definite clinical advantages. However, in elderly patients undergoing laparoscopic surgery, hypercapnia caused by pneumoperitoneum establishment and neuropathic pain caused by direct surgical stimulation can both induce reflexivity and increase sympathetic activity during surgery.^3^ At the same time, there may also postoperative stress disorder and cognitive impairment.^4^ In this regard, for elderly patients undergoing laparoscopic surgery, it is particularly important to select the appropriate anesthesia to maintain the most stable and perfect anesthetic potency.^5^

Both sevoflurane and propofol are relatively broad-spectrum ultrashort acting anesthetics, which have significant advantages in anesthetic potency and safety.^6^,^7^ Previous studies have documented that sevoflurane inhalation combined with intravenous propofol anesthesia may achieve better anesthetic benefits.^8^ Simultaneously, both anesthetics can also produce an effect on the coagulation-related functions of the body through the inhibition of stress response, anti-platelet aggregation, anti-inflammation, antioxidation and other mechanisms.^9^,^10^ However, at present, there are few studies focusing on the effects of the proposed mode of anesthesia on coagulation function, postoperative stress disorder and cognitive function in elderly patients undergoing laparoscopic surgery. In view of the above, the present study was carried out prospectively with the collection of eligible cases to provide high-value clinical reference opinions for general anesthesia of elderly patients undergoing laparoscopic surgery.

## METHODS

Eligible subjects were collected from the elderly patients who planned to undergo laparoscopic surgery in Xianning Central Hospital from September 2014 to February 2018. According to the corresponding inclusion and exclusion criteria, eighty cases were selected and were randomly divided into observation group and control group based on the random regionalization method, with 40 cases in each group. In the observation group, there were 21 males and 19 females, with an average age of (71.82±2.13) years old (65~79 years old) and an average weight of (59.24±6.13) kg (49~73 kg). There were 18 cases of grade I and 22 cases of Grade-II in accordance with the grading standard of American Society of Anesthesiologists (ASA) before operation. Besides, there were 11 cases of laparoscopic cholecystectomy, ten cases of laparoscopic repair of acute perforated duodenal ulcer, 12 cases of laparoscopic ureterolithotomy and seven cases of laparoscopic hysterectomy, with the operative time of (147.25±35.12)minutes. While in the control group, there were 25 males and 15 females aged from 65 to 80 years and weighed from 47 to 72 kg, with an average of 72.04±3.11) years old and (58.24±5.71) kg, respectively. Twenty-one patients showed preoperative ASA Grade-I and 19 cases of Grade-II. In addition, there were 10 cases of laparoscopic cholecystectomy, 12 cases of laparoscopic repair of acute perforated duodenal ulcer, 10 cases of laparoscopic ureterolithotomy and 8 cases of laparoscopic hysterectomy, with the operative time of (150.33±34.87) min. No statistical difference was found in gender, age, weight, operation type and operative time between the two groups (p>0.05).

### Ethical Approval

The study was approved by the Institutional Ethics Committee of Xianning Central Hospital, The First Affiliated Hospital of Hubei University of Science and Technology on 10 April, 2021(No.:2021016), and written informed consent was obtained from all participants.

### Inclusion criteria:

- Patients undergoing elective laparoscopic surgery.
- Patients aged >65 years old.
- Patients with preoperative ASA grade I~II;
- Patients without risk factors for thrombosis included obesity, hypertension, coronary heart disease and diabetes.
- Patients without severe uncontrollable cardiovascular and cerebrovascular diseases. All patients or family members were aware of the study content and signed corresponding informed consent.

### Exclusion Criteria

- Patients with preoperative ASA grade III or above.
- Patients with severe anemia (Hb<110g/L)
- Patients with medical history of opioid allergy.
- Patients with anticoagulant use during perioperative period.
- Patients with acute and chronic respiratory disease history;
- Patients with history of drug or alcohol abuse.

After entering the room, patients were provided with the establishment of the dorsal hand vein channel, monitoring of electrocardiograph, pulse oxygen saturation and noninvasive blood pressure. Then, patients were supplied with injection of Penehyclidine Hydrochloride (Chengdu List Pharmaceutical Co., Ltd., H20020606, 0.01 mg/kg), and pumping of Dexmedetomidine Hydrochloride (Sichuan Guorui Pharmaceutical Co., Ltd., H20110097, 1 μg/kg) intravenously within 10min. Anesthesia was induced by Midazolam (Yichang Humanwell Pharmaceutical Co., Ltd., H20067040, 0.04 mg/kg) + Atracurium Besylate (Shanghai Hengrui Pharmaceutical Co., Ltd., H20061298, 0.3 mg/kg) + Fentanyl Citrate (Jiangsu Nhwa Pharmaceutical Co., Ltd., H20113508, 5 μg/kg) + Etomidate Fat Emulsion (Jiangsu Nhwa Pharmaceutical Co., Ltd., H20020511, 0.25 mg/kg) in both groups. An anesthetic ventilator was connected after intubation, with fresh gas flow of 2 L/minutes, respiratory rate of 12 times/min, tidal volume of 6-8 ml/kg, and inspiratory-to-expiratory ratio of 1:2 under the volume control mode. Invasive hemodynamic test was performed by radial artery puncture. Furthermore, anesthesia was maintained in the control group with continuous intravenous infusion of propofol at a dose of 50~200 μg/(kg·minutes), and simultaneous intravenous pumping of Atracurium Besylate (1 mg/kg·hour). While in the observation group, an additional 1-2% sevoflurane inhalation was used to maintain anesthesia on the basis of the scheme in the control group. Meanwhile, 2 μg/mg Fentanyl Citrate was added before skin incision in the two groups. Intraoperatively, propofol dosage and sevoflurane inhalation concentration were adjusted according to Narcotrend Index (NI) to maintain the index between D1 and E1. After the operation, patients were transferred to the Anesthesia Recovery Room and given Prostigmin Methylsulfate (40 μg/kg) and Atropine Sulphate (20 μg/kg) after the recovery of spontaneous breathing. The patients were observed with active swallowing and cough reflex, unobstructed breathing, stable hemodynamics after 5 minutes of air inhalation, after which the endotracheal tube was pulled out and patients were returned to the Inpatient Ward. All patients were supplied with self-control analgesic pump for analgesia,with the use of Sufentanil (2 μg/kg) + Dezocine (20 mg) + Tropisetron (10 mg) + 0.9% normal saline to 100ml for patient-controlled intravenous analgesia (PCIA). The resting Visual Analog Scale (VAS) score was maintained at or below 2 at rest, and below 3 during physical activity.

### Observed Indicators:

### General data of the two groups

Intraoperative blood transfusion ratio, complications, anesthesia induced unconsciousness time, intubation time, pneumoperitoneum establishment time, spontaneous breathing recovery time, eye opening time upon calling and extubation time.

### Intraoperative hemodynamics

Heart rate (HR), systolic blood pressure (SBP) and mean arterial pressure (MAP) of the two groups when entering the room, before intubation, 0 minutes, three minutes and six minutes after intubation, 0min and 5min after pneumoperitoneum establishment, and during exploration;

### Postoperative stress disorder

Five dimensions of subjective evaluation of traumatic time, re-experienced symptom, avoidance symptom, increased alertness and impaired social function based on by PTSD-SS scale, with a total of 24 items. According to the score of impact degree, scores were ranged between from no impact to significant impact. A higher the score might indicate a more serious stress disorder;

### Postoperative cognitive function

Multiple dimensions such as orientation, memory, attention and calculation, memory ability, language ability, etc. by using the Mini Mental State Examination (MMSE). A higher score might suggest a better cognitive function.

### Statistical Analysis

SPSS19.0 software was used for statistical analysis. Counting data were expressed by cases (n) and compared by using χ^2^ test. The measurement data such as general surgical data, hemodynamics, postoperative stress disorder score and cognitive function scores were expressed in ±s. The paired t-test was used for pairwise comparison within the group, and the paired t-test for inter-group comparison. p<0.05 meant that the difference was statistically significant.

## RESULTS

There were no blood transfusions and no complications during operation in the two groups, and the operation completed successfully. As shown in Table-I, there was no significant difference in the time of unconsciousness, intubation time, and duration of pneumoperitoneum establishment (p>0.05). However, the spontaneous breathing time, eye opening time upon calling and extubation time in observation group were shorter than those in control group (p<0.05).

**Table-I:** Comparison of general data of surgery between groups (*x̅*±*s*).

Groups	Time of unconsciousness (min)	Intubation time (min)	Duration of pneumoperitoneum (min)	Quality of recovery from anesthesia		

				Spontaneous breathing time (min)	Eye opening time upon calling (min)	Extubation time (min)
Observation group	1.76±1.03	4.17±1.85	119.47±29.84	8.97±1.15	12.67±3.37	14.70±4.68
Control group	1.85±1.10	4.15±1.83	119.45±30.01	9.64±1.64	14.58±1.33	17.33±4.50
t	0.377	0.048	0.002	2.115	2.642	2.561
p	0.706	0.961	0.997	0.037	0.010	0.012

The HR in the two groups at intubation and at exploration was significantly higher than that at the time of entering the room (p<0.05), which was lower in observation group than that in control group (p<0.05). While there was no significant difference in the index at the other time points compared with that before entering the room and control group (p>0.05). The SBP had different degrees of change before intubation, after 0min, three minutes and six minutes of intubation, after 0 minutes and five minutes of pneumoperitoneum establishment and at exploration when compared with that before entering the room (p<0.05). But the SBP at intubation and at exploration was significantly lower in observation group than that in control group (p<0.05). MAP was only decreased significantly at intubation compared with that before entering the room (p<0.05). At exploration, there was no significant fluctuation in the MAP level in observation group (p>0.05), but the MAP was increased significantly in control group (p<0.05). Corresponding results were shown in Table-II.

**Table-II:** Comparison of intraoperative hemodynamic indexes between groups (*x̅*±*s*).

Hemodynamic indexes	At the time of entering the room	Before intubation	During intubation			During pneumoperitoneum establishment		At exploration

			0min	3min	6min	0min	5min	
HR (f/min^-1^)	-	-	-	-	-	-	-	-
Observation group	70.45±10.32	65.38±8.29	73.44±9.37^①^	64.12±10.17	64.10±10.12	69.06±10.13	69.43±10.76	77.12±11.25^①^
Control group	68.33±9.41	65.35±7.17	80.11±9.96^①^	68.44±11.43	68.11±9.32	68.41±9.44	67.12±10.33	85.45±^①^7.64
t	0.507	0.017	3.084	1.785	1.843	0.296	0.980	3.874
p	>0.05	>0.05	<0.05	>0.05	>0.05	>0.05	>0.05	<0.05
SBP (mmHg)	-	-	-	-	-	-	-	-
Observation group	126.09±9.42	110.33±10.47^①^	119.25±7.61^①^	109.45±7.20^①^	111.36±10.24^①^	109.38±6.47^①^	115.45±6.09^①^	125.33±9.28
Control group	124.35±7.12	110.66±8.15^①^	135.97±6.82^①^	111.41±9.27^①^	109.82±10.33^①^	111.64±7.52^①^	117.33±10.65^①^	136.27±7.81^①^
t	0.931	0.157	10.348	1.056	0.669	1.440	0.969	5.704
p	>0.05	>0.05	<0.05	>0.05	>0.05	>0.05	>0.05	<0.05
MAP (mmHg)	-	-	-	-	-	-	-	-
Observation group	90.47±5.02	80.33±6.45^①^	88.37±6.10	86.41±5.32	84.12±5.09	85.33±5.01	87.12±5.69	91.20±4.36
Control group	92.17±5.36	82.44±6.10^①^	91.01±6.37	86.12±4.05	84.66±7.12	85.45±5.09	89.31±5.70	97.31±6.29^①^
t	1.464	1.503	1.893	0.274	0.274	0.106	1.719	5.875
p	>0.05	>0.05	>0.05	>0.05	>0.05	>0.05	>0.05	<0.05

***Note:*** Compared with the value at the time of entering the room,

① p<0.05.

As listed in Table-III, the scores of subjective assessment, re-experienced symptom, avoidance symptom, increased alertness and impaired social function were significantly lower in the observation group than those in the control group, with statistical differences (p<0.05).

**Table-III:** Comparison of postoperative stress disorder between groups (*x̅*±*s*, points).

Groups	Subjective assessment	Re-experienced symptom	Avoidance symptom	Increased alertness	Impaired social function
Observation group	6.06±1.39	4.71±1.37	2.79±1.55	3.31±1.17	2.62±1.65
Control group	7.57±2.22	5.40±1.35	4.63±1.75	4.29±1.49	3.41±1.31
t	3.646	2.268	4.97	3.271	2.371
p	<0.05	<0.05	<0.05	<0.05	<0.05

There was no significant difference in cognitive function between the two groups before operation (p>0.05). At one hour, three hour, six hour and twenty four hour after operation, the cognitive function of the two groups decreased to varying degrees, which, however, was significantly higher in the observation group than that of the control group, and the difference was statistically significant (Table-IV).

**Table-IV:** Comparison of postoperative cognitive function between groups (*x̅*±*s*, points).

Groups	Before operation	1h after operation	3h after operation	6h after operation	24h after operation
Observation group	29.72±0.41	25.95±1.96^①^	26.97±1.60^①^	27.29±1.33^①^	28.79±1.44^①^
Control group	29.65±0.43	24.23±1.62^①^	25.18±1.52^①^	25.81±1.39^①^	27.16±1.31^①^
t	0.745	4.277	5.129	4.865	5.29
p	>0.05	<0.05	<0.05	<0.05	<0.05

Note: Compared within group before operation,

① p<0.05.

## DISCUSSION

Sevoflurane and propofol are widely used broad-spectrum anesthetics in the clinical setting. Sevoflurane is a new volatile inhalational anesthetic agent, which is also an inhalation anesthetic with the least hepatotoxicity.^11^ It can not only play the role of anesthesia induction in a very short time, but also has small blood/air partition coefficient, no irritation to respiratory tract and only mild inhibition to the circulatory system.^12^ While propofol is a short-acting intravenous anesthetic. Previous studies have reported that it has a short duration of action, a strong sedative and amnesiac effect, and a high quality of consciousness.Besides, the orientation of patients may not be affected after waking, accompanied by a low incidence of postoperative side effects such as nausea and vomiting.^13^ However, the analgesic effect of propofol has not been fully clarified. According to previous research, when used alone, it may produce relatively poor analgesic effect, requires large dosage and hence high cost, which may also result in violent fluctuation of the hemodynamics due to the excessive dosage.^14^ Simultaneously, due to the possible inhibition of Na+ influx, it may further inhibit the depolarization of presynaptic membrane, which may lead to the release of glutamate, enhance GABA postsynaptic action, and causes the loss of consciousness, disappearance of somatic motor reflex, memory function and other adverse anesthetic effects.^15^

In recent decades, compound anesthesia has been paid much attention to during clinical process. It can not only make use of the synergy between drugs, but also reduce the dosage of anesthetics relatively, thereby reducing the risk of dose-related adverse reactions and ensuring more stable and perfect anesthesia benefits. Among them, combined intravenous and inhalation anesthesia is a typical representative of balanced anesthesia.^16^ It can give full play to the advantages of inhalation anesthesia and intravenous anesthesia, and can effectively control surgery-induced cardiovascular reflex without obvious circulatory inhibition, which is more conducive to maintaining an appropriate depth of anesthesia.^17^ Simultaneously, the use of two anesthetic agents have fast metabolism and excretion without accumulation, which avoids the defect of long-lasting duration of propofol anesthesia alone, thereby improving the quality of post-anesthesia wake-up and facilitating postoperative recovery of consciousness significantly.^18^ In our study, there was no significant difference in the time of unconsciousness, intubation time, and duration of pneumoperitoneum establishment. However, the spontaneous breathing time, eye opening time upon calling and extubation time in observation group were shorter than those in control group. These results suggested that compared with propofol alone, sevoflurane combined with propofol can improve the recovery quality from anesthesia, which is consistent with previous reports.^19^

Meanwhile, in our study, the HR levels of the two groups immediately after intubation and during exploration were significantly higher than those at the time of entering the room. While the level in the observation group was lower than that of the control group, with statistically significant difference, and there was no significant difference at other time points compared with that at the time of entering the room and the control group. The SBP had different degrees of increase or decrease before intubation, after zero three and six minutes of intubation, after 0min and 5min of pneumoperitoneum establishment and at exploration when compared with that before entering the room. However, the SBP at intubation and at exploration was significantly lower in observation group than that in control group, with statistically significant difference. Moreover, MAP showed a significant decrease merely at intubation compared with that before entering the room. At exploration, there was no significant fluctuation in the MAP level in observation group, but the level was increased significantly in control group, with statistically significant difference. These results suggest that propofol, as well as sevoflurane combined with propofol have slight effects on HR, SBP and MAP in elderly patients undergoing laparoscopic surgery.

Except obvious fluctuation of HR at intubation and during exploration, SBP before intubation, at different time points of intubation and pneumoperitoneum establishment, MAP during intubation, there was no significant difference in HR, SBP and MAP at other time points from those at the time of entering the room, There was more evident fluctuation range of the control group. These data support that sevoflurane combined with propofol and propofol alone may have slight impact on hemodynamics in elderly patients undergoing laparoscopic surgery, with less impact by using the compound anesthesia. It is consistent with the report of Han Y et al.^20^ At the same time, the scores of dimensions including subjective assessment, re-experienced symptom, avoidance symptom, increased alertness and impaired social function in MMSE scale were significantly lower postoperatively in the observation group than those in the control group. It is suggested that there may be a milder degree of postoperative stress disorder in the observation group. In addition, the cognitive function of the observation group at any time point after operation was better than that of the control group.

### Limitations of the study:

Cautiously, in view of the relatively small sample size of this study, there is a need to further confirm the effects of sevoflurane combined with propofol on intraoperative hemodynamics, postoperative stress disorder and cognitive function in elderly patients undergoing laparoscopic surgery, so as to offer continuous replenishment and improvement.

## CONCLUSION

Intravenous anesthesia with sevoflurane combined with propofol has little effects on intraoperative hemodynamics and postoperative cognitive function in elderly patients undergoing laparoscopic surgery. Besides, it results in milder postoperative stress disorder than use of propofol alone.

### Authors’ Contributions:

**LY & DX:** Designed this study, prepared this manuscript,d are responsible and accountable for the accuracy and integrity of the work.

**DX:** Collected and analyzed clinical data.

**ZC:** Daxta analysis, Significantly revised this manuscript.
